# Discharge profile of a zinc-air flow battery at various electrolyte flow rates and discharge currents

**DOI:** 10.1038/s41597-020-0539-y

**Published:** 2020-06-22

**Authors:** Ali Abbasi, Soraya Hosseini, Anongnat Somwangthanaroj, Rongrong Cheacharoen, Sorin Olaru, Soorathep Kheawhom

**Affiliations:** 1 Department of Chemical Engineering, Faculty of Engineering, Chulalongkorn University, Bangkok, Thailand; 2 Metallurgy and Materials Science Research Institute, Chulalongkorn University, Bangkok, Thailand; 3 Laboratoire des signaux et systèmes, Université Paris-Saclay, CNRS, CentraleSupélec, Gif-sur-Yvette, France; 4 Research Unit of Advanced Materials for Energy Storage, Chulalongkorn University, Bangkok, Thailand

**Keywords:** Batteries, Chemical engineering

## Abstract

Nowadays, due to global warming stemming from excessive use of fossil fuel, there is considerable interest in promoting renewable energy sources. However, because of the intermittent nature of these energy sources, efficient energy storage systems are needed. In this regard, zinc-air flow batteries (ZAFBs) are seen as having the capability to fulfill this function. In flow batteries, the electrolyte is stored in external tanks and circulated through the cell. This study provides the requisite experimental data for parameter estimation as well as model validation of ZAFBs. Each data set includes: current (mA), voltage (V), capacity (mAh), specific capacity (mAh/g), energy (Wh), specific energy (mWh/g) and discharge time (h:min:s.ms). Discharge data involved forty experiments with discharge current in the range of 100–200 mA, and electrolyte flow rates in the range of 0–140 ml/min. Such data are crucial for the modelling and theoretical/experimental analysis of ZAFBs.

## Abstract (summary)

**Table:** 

Measurement(s)	electrical current • Voltage • battery capacity • specific discharge capacity • energy • specific energy • discharge time
Technology Type(s)	battery testing system
Factor Type(s)	electrolyte flow rate • discharge current

Machine-accessible metadata file describing the reported data: 10.6084/m9.figshare.12423878

## Background & Summary

Of late, owing to technological advancements and population growth, demand for energy has increased dramatically. The majority of this energy demand has been supplied by fossil fuel consumption. Consequently, this has led to the release of huge amounts of greenhouse gases into the atmosphere giving rise to issues, such as global warming^1^. To tackle this issue, renewable energy sources have been greatly encouraged. However, because of the erratic and random nature of renewable energy sources viz. solar and wind energy, the development of more effective and reliable energy storage devices is vital. Batteries are deemed as one of the most efficient technologies to fill the time gap between energy production and consumption^2^.

As promising energy storage systems, metal-air batteries have gained widespread attention. In fact, as potential alternatives for lithium-ion batteries (LIBs), zinc-air batteries (ZABs) are highly regarded. ZABs offer high theoretical energy density of 1086 Wh/kg (including oxygen) and 1350 Wh/kg (excluding oxygen) which is five times higher than that of current LIBs^3–5^. Furthermore, ZABs have a simple design, low operational temperature, high efficiency, high safety and are environmentally friendly^6,7^. Compared to other metal anodes, zinc is an inexpensive, abundant and non-toxic element with greater stability in aqueous environments^8–10^.

Before they can be fully commercialized, ZABs face many challenges that need to be overcome. These batteries usually use highly alkaline electrolytes. When the alkaline electrolytes get in touch with CO_2_ in the air, they form carbonates that limit the lifetime of the battery. Using pH-buffered near-neutral electrolytes instead of alkaline solutions is one of the methods to reduce carbonate absorption. However, most of near-neutral electrolytes contain corrosive halide salts which threaten the formation of zinc oxide (ZnO) as the main product of anode oxidation. Clark *et al*.^11^ designed halide-free aqueous electrolytes for ZABs using thermodynamic descriptors for computational screening components. They simulated dynamic performance of one possible halide-free aqueous electrolyte in a ZAB using an advanced method of continuum modeling and validated the results by experiments.

In electrically rechargeable ZABs, a bifunctional air electrode for oxygen reduction reaction (ORR) during discharge mode and for oxygen evolution reaction (OER) during charge mode is required. However, owing to the lack of a suitable bifunctional catalyst, mainly three-electrode configurations with separate charge and discharge air electrodes are used^12^. Nonetheless, a two-electrode configuration is preferred due to its simplicity and more compact design. Various bifunctional catalysts have been reported in the literature, such as, La_0.6_Sr_0.4_Co_0.2_Fe_0.8_O_3_ perovskite catalyst^13^ and CCOP_TDP_–FeNi–SiO_2_ derived from predesigned covalent organic polymers^14^.

There are other objections in the way of developing ZABs: their low round trip efficiency due to oxygen overpotential, air cathode flooding leading to low durability, non-uniform zinc plating/dendrite formation^15^, and a lack of highly conductive and selective separator to minimize zincate cross-over^6^.

ZABs can be fabricated in various shapes and designs such as flexible batteries^16–18^, cable type batteries^19^ and flow batteries^20,21^. In flow batteries, high depth of discharge is possible which means most of its nominal capacity can be discharged without imposing any permanent damage to the cell structure^22^. In addition, they can store electroactive materials required for battery operation in a tank outside the battery structure. It provides higher flexibility regarding energy and power decoupling^23^, and lower mechanical stress on the electrodes in comparison with other types of batteries, such as LIBs, leading to the fabrication of long-lasting systems^24^. As a result, the scale-up of flow batteries can be achieved in a cost-effective way for stationary large-scale applications^25^. A disadvantage is the reduced energy efficiency due to the required electrolyte movement^24^.

Unlike pure flow batteries such as vanadium redox flow batteries (VRFB), ZAFBs with a zinc anode inside the battery, are deemed as hybrid flow batteries. In ZAFBs, power and energy are not completely decoupled. Energy stored in the battery depends on the amount of zinc available in the anode^15^.

Some experimental studies have focused on various aspects of ZAFBs. For instance, Zelger *et al*.^24^ studied the diverse physico-chemical properties of sodium hydroxide (NaOH) and potassium hydroxide (KOH) solution electrolytes, including refractive index, conductivity, density of electrolytes as well as the rest potential of zinc electrodes in these solutions, as indicators of the state of charge (SOC). In another study, a filtration system was used for the circulating electrolyte to remove zinc oxide from the saturated electrolyte. Thus, this enabled the size of the required electrolyte and storage tank to be decreased dramatically, leading to an increase in energy density of the cell^26^. Further, Kupsch *et al*.^27,28^ introduced a new method for characterizing local flow conditions in a highly laden zinc suspension used for a ZAFB; thereby, increasing the accuracy of measurements. To prevent dendrite formation during the charging mode of ZAFBs, a trapezoidal structure or a permanent magnet parallel to an anode has been proposed^29^. Pichler *et al*.^25^ studied the effects of several parameters including flow rate, electrolyte concentration, and current density on the performance and stability of electrodes.

For the development of ZABs, model-based engineering has proved to be very effective. It offers better understanding of the behavioural characteristics of the batteries, resulting in an enhanced design and operation^30–32^. Nevertheless, it is acknowledged that developed models need to be validated using reliable experimental data, in various configurations. In a study provided by MATSI Inc., experimental data were used to confirm the validity of a model developed by researchers on a primary ZAB^33^. Besides, Deiss *et al*.^34^ set up a numerical model to simulate the cycle performance of a ZAB. The model was validated using experimental data produced in their respective laboratory premises. Furthermore, Schröder and Krewer^35^ used the same experimental data to validify their isothermal mathematical model of a rechargeable ZAB having an alkaline liquid electrolyte. More recently, a mathematical model was introduced for analyzing the performance of an integrated ZAB/zinc electrolyzer^30^. This model was undertaken to evaluate the influence of operating parameters on the system efficiency, regarding hydrogen evolution reaction (HER). The model was validated against experimental data obtained from an in-house developed ZAB and a zinc electrolyzer. Yang *et al*.^36^ optimized parameters affecting zinc reduction during battery charging using the Taguchi method. Parameters included: electrolyte temperature, pulse current, pulse frequency, and duty cycle. In another study, a computational fluid dynamic (CFD) model was developed for simulating physical and chemical processes in a ZAFB. In a comparison of both numerical and experimental values, it was noted that the data proved to be in good agreement with the predicted and measured values of the voltage–current data^9^. Amunategui *et al*.^15^ developed a mechanical model to better understand the shunt current in a pilot-scale ZAFB and the model was validated by experimental results.

Investigations regarding model-based engineering of ZABs and ZAFBs are still in the early stages. Such studies require further research and publicly available data. Likewise, the availability of more reliable experimental data will enhance the model-based engineering of the batteries. This will lead to more accurate models which are required for large-scale applications. Previously, experimental data^37^ along with data description^38^ of a tubular designed non-flow ZAB were published. Such data involved discharge profiles at different constant discharge currents, and dynamic behavior at different step changes of discharge current. While the data were acquired from a non-flow ZAB, the data mainly supported dynamic modelling for control and operational purposes.

Herein, the main aim of this work is to provide experimental data of a ZAFB. Such data include: discharge profiles of a ZAFB at various constant discharge currents and electrolyte flow rates. Actually, a tubular ZAFB was developed in-house and was consequently used to obtain all the testing data. Thus, the data provided in this study could be used to validate mathematical models on ZAFBs and approximate the variables for empirical models. Moreover, the data could be used to support the design and analysis of the effects of electrolyte flow rates and discharge currents.

## Structure, Construction and Measurement Methodology

### Chemical and materials

Ethanol and toluene (Grade AR) were supplied by QReC. A nickel (Ni) foam cathode current collector, 1 mm thick, 99.97% purity and 100 pores per inch (PPI) porosity, was purchased from Qijing Trading Co., Ltd. Both an anode current collector and a cell chamber structure were made of SUS 304 stainless steel mesh (20 and 30 mesh), respectively, supplied by Alikafeii Trading Co., Ltd. Zinc granules (Sirikul Engineering Ltd.; 20 mesh, 99.99% purity), carbon black (Vulcan® XC-72, Cabot Corporation), and BP-2000 (BLACK PEARLS® 2000, Cabot Corporation) were used as received. Poly (styrene-co-butadiene) for preparation of the cathode binder was purchased from Sigma-Aldrich. The cell separator was prepared using poly(vinyl acetate) (PVAc) from TOA Paint Public Co., Ltd. and Whatman filter paper (No. 4, Sigma-Aldrich). Industrial grade potassium hydroxide (KOH) pellets, 99% purity, purchased from CT Chemical Co., Ltd., were used to prepare the electrolyte solution. Polytetrafluoro-ethylene (PTFE) powder (1 μm diameter) and manganese dioxide (MnO_2_) powder (5 µm diameter) from Sigma-Aldrich, were used to prepare the air cathode.

### Battery fabrication and operation

The configuration of a ZAFB is similar to a stagnant battery. The battery is comprised of: a zinc anode (with anode current collector), a cathode (with gas diffusion layer, catalyst layer and cathode current collector), a separator (placed between the anode and the cathode) and the electrolyte solution (filling the gap between the anode and the separator). The main difference is that in a flow battery, the electrolyte is pumped from an external electrolyte tank and is circulated with a controlled flow rate in the battery, mainly to suppress anode passivation^26^. When the battery is discharging, the anode is oxidized, according to the following equations:

1 $${\rm{Zn}}+4{{\rm{OH}}}^{-}\to {\rm{Zn}}{({\rm{OH}})}_{4}^{2-}+2{{\rm{e}}}^{-}$$

2 $${\rm{Zn}}{({\rm{OH}})}_{4}^{2-}\to {\rm{ZnO}}+{{\rm{H}}}_{2}{\rm{O}}+2{{\rm{OH}}}^{-}$$

3 $${\rm{Total}}\,{\rm{Anode\; :}}\,{\rm{Zn}}+2{{\rm{OH}}}^{-}\to {\rm{ZnO}}+{{\rm{H}}}_{2}{\rm{O}}+2{{\rm{e}}}^{-}$$

In the cathode, oxygen from the air receives the electrons released in the anode through an external circuit and is reduced to hydroxide ions, as in Eq. (4):

4 $$1/2\,{{\rm{O}}}_{2}+{{\rm{H}}}_{2}{\rm{O}}+2{{\rm{e}}}^{-}\to 2{{\rm{OH}}}^{-}$$

There are two typical configurations for ZAFBs: a square flat design^26,29^ and a tubular design^21,36,39^. In both designs, the order of component placements in both designs is similar. However, in the flat configuration, the anode, cathode and separator are arranged as parallel sheets. In the tubular design, the components are placed in a concentric form around a central tube.

**Fig. 1:**
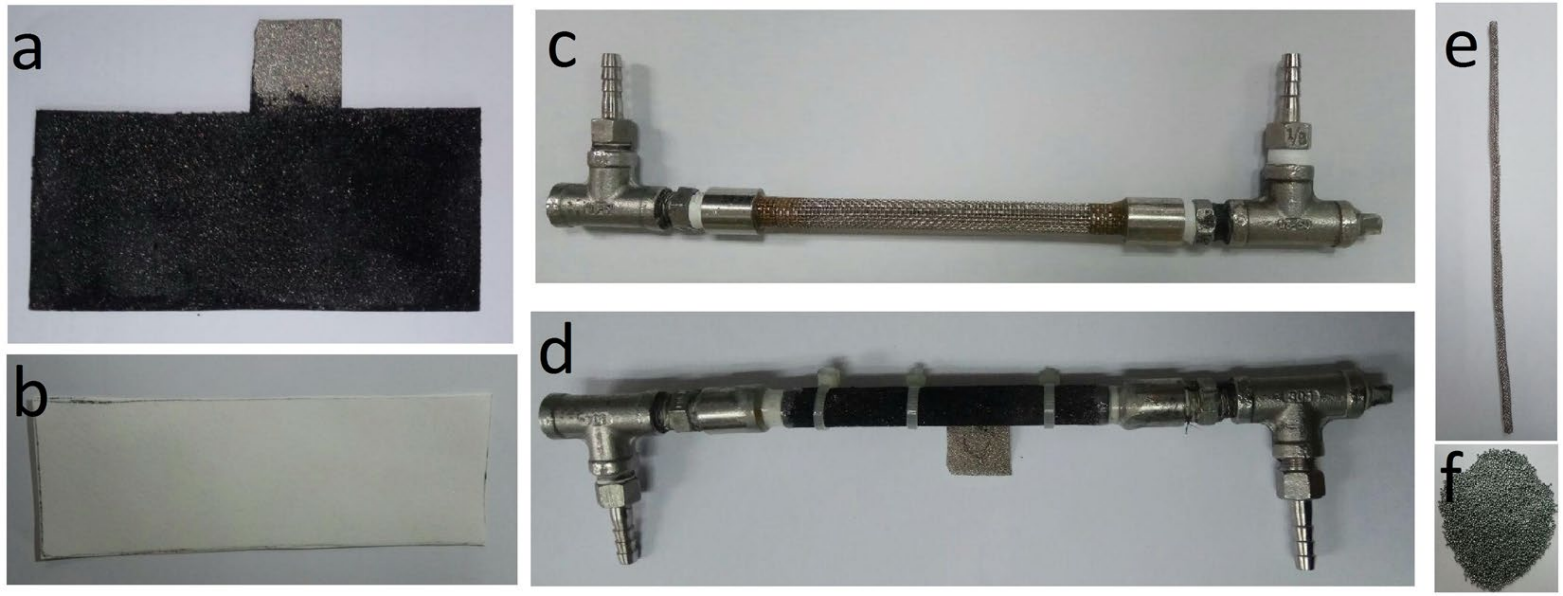
In-house developed ZAFB: (**a**) air cathode (**b**) separator (**c**) battery’s stainless-steel framework (**d**) final assembled battery (**e**) tubular anode current collector and (**f**) zinc granules (anode active material).

For battery tests, the in-house developed tubular ZAFB was used. Figure 1 shows the components required for the cell assembly and completed cell. The main structure of the cell was made of stainless-steel mesh wrapped in a tubular shape with T-shaped connections at both ends for electrolyte circulation (Fig. 1c). The separator membrane was made of Whatman filter paper (No. 4) which was coated with 24 wt.% PVAc solution providing a final thickness of 200 µm (Fig. 1b). It was wrapped, as the first layer, around the stainless-steel mesh. The air cathode made of Ni-foam, coated with various components on both sides (Fig. 1a), was wrapped on top of the separator. All layers were fixed in place using cable ties and PVAc glue was used to seal the battery to minimize electrolyte leakage (Fig. 1d).

To prepare the cathode, Ni-foam was used as the current collector and gas diffusion layer. A mixture of BP-2000 and PTFE (30:70 wt.%) dispersed in ethanol was coated on one side of the Ni-foam (gas diffusion side) and pressed using a hot-press at 350 °C for 15 min. To prepare the inner (active catalyst) side of the Ni-foam, a mixture of MnO_2_, BP-2000 and VXC-72 (30:35:35 wt.%) was used. The mixture was stirred in toluene for 1 h. and after adding polystyrene-co-butadiene as the binder, stirring continued for another hour. Finally, the inner side of the Ni-foam was coated with the mixture and pressed using a hot-press at 150 °C for 10 min. Figure 1a shows the final cathode with an active area of 31.4 cm^2^ and catalyst loading of 2.7 mg/cm^2^. It has a 2 × 2 cm uncoated tongue on one side, acting as the connection point of the electrode.

The anode current collector was a tubular stainless-steel mesh (30 mesh), 0.5 cm in diameter (Fig. 1e), filled with 5 g of 20 mesh zinc granules as active anode material (Fig. 1f). By using the same amount of zinc granules in all experiments, the maximum capacity of the batteries is controlled; thus making the final values of specific capacity and specific energy comparable. The tube was placed in the center of the cell and was connected electrically to the battery mainframe on both sides. Before putting the anode inside and discharging the battery, the cell was filled with 7 M KOH, for 2 h. to saturate the separator.

**Fig. 2:**
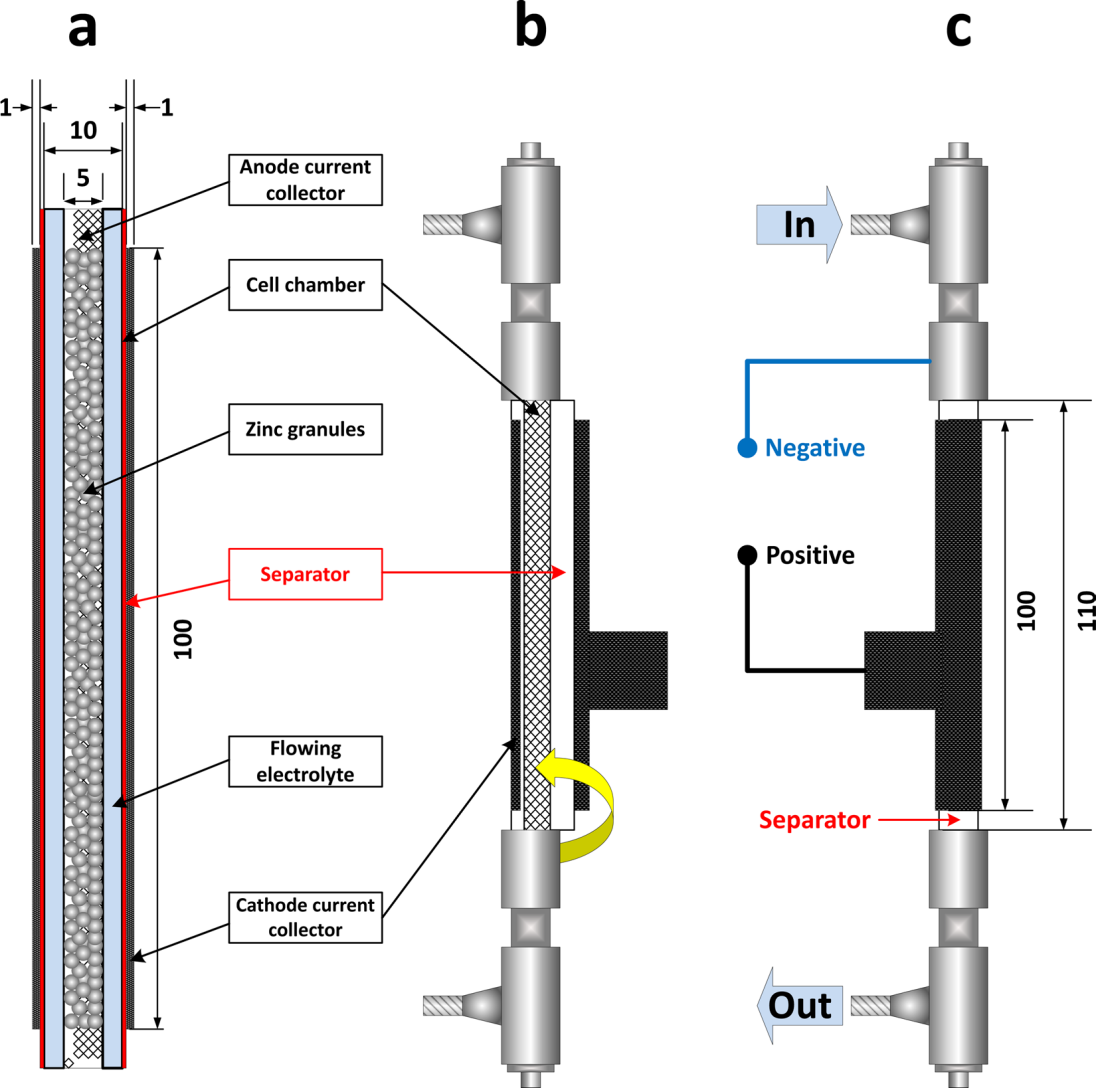
Schematic view of (**a**) cross-section of the active middle part of the battery (**b**) battery fabrication by wrapping the separator and cathode current collector around the cell chamber and (**c**) final structure of the in-house developed ZAFB with electrolyte Inlet/Outlet.

In Fig. 2, a schematic view and dimensions of the cell is shown. 7 M KOH solution was used as the electrolyte, circulating through the cell during its operation via a peristaltic pump, in a flow rate range of 0–140 ml/min. It is noted that changing flow rate results in a change in the pressure of the electrolyte inside the cell which may change the wetting depth of the air cathode. This is one of the effects of changing the flow rate which is reflected in the final specific capacity and specific energy. However, the effect of electrolyte flow rate on the wetting depth of the air cathode was not evaluated independently in this study. The cell was discharged in a discharge current range of 100–200 mA. Table 1 shows the cell operating conditions for various runs as well as the name of the corresponding data file. A summary of the cell assembly components, along with the quantities/parameters used, is shown in Table 2.

**Table 1:** Cell operation condition for various runs and corresponding data file names.

Electrolyte flow rate (ml/min)	Discharge Current (mA)				
	100	125	150	175	200
20	EXP-1	EXP-2	EXP-3	EXP-4	EXP-5
40	EXP-6	EXP-7	EXP-8	EXP-9	EXP-10
60	EXP-11	EXP-12	EXP-13	EXP-14	EXP-15
80	EXP-16	EXP-17	EXP-18	EXP-19	EXP-20
100	EXP-21	EXP-22	EXP-23	EXP-24	EXP-25
120	EXP-26	EXP-27	EXP-28	EXP-29	EXP-30
140	EXP-31	EXP-32	EXP-33	EXP-34	EXP-35
0	EXP-36	EXP-37	EXP-38	EXP-39	EXP-40

**Table 2:** Summary of cell components and parameters used.

Components	Material/Parameters
Anode active material	5 g zinc granules (20 mesh) packed inside anode current collector tube
Anode current collector	0.5 cm-diameter tubular stainless-steel mesh (20 mesh)
Cathode active material	Oxygen in the atmospheric air
Catalytic layer	a mixture of MnO_2_ (30 wt.%), BP-2000 (35 wt.%) and VXC-72 (35 wt.%) (catalyst loading of 2.7 mg/cm^2^); Binder: polystyrene-co-butadiene, 5 wt.% of dry mixture
Gas diffusion layer	A mixture of BP-2000 (30 wt.%)/PTFE (70 wt.%)
Cathode current collector	Ni-foam, 1 mm thick and 10 cm in length, having an active surface area of 31.4 cm^2^
Separator	Whatman filter paper (No. 4) coated with 24 wt.% polyvinyl acetate solution, having a final thickness of 200 µm
Electrolyte	150 ml of 7 M KOH solution circulating through the cell

### Measurement and data collection

Discharge performance of the batteries was evaluated using battery testing system (NEWARE BTS-4000 series, Neware Technology Ltd., China). Room temperature was controlled in the range of 25–27 °C using an air conditioner. The relative humidity, oxygen content, and CO_2_ content of the room were 70%, 20.8 v/v%, and 412 ppm, respectively. The battery was placed in a box having some holes in it for air circulation, to minimize environmental fluctuations. The cell was discharged at a constant discharge current in the range of 100–200 mA and the data was logged every second by BTSDA (Battery Testing System Data Analyzer) software, version 7.6.0.124. Output data was in the format of Excel file, containing various information, including: current (mA), voltage (V), capacity (mAh), energy (Wh), and time (h:min:s.ms). A discharge profile test was carried out via discharging the battery at a constant discharge current, until the battery was exhausted. Two extra columns of specific capacity (mAh/g) and specific energy (mWh/g) was added to the output Excel file to incorporate the weight of the zinc granules in the final values.

## Data Records

Each Excel file provides the output data. Thus, each file contains the discharge profile of the battery, at different constant discharge currents, in the range of 100–200 mA and various electrolyte flow rates in the range of 0–140 ml/min. Tests to determine the range of discharge current and electrolyte flow were conducted and showed that when discharge current increased more than 200 mA, it led to instability in the discharge voltage. Furthermore, electrolyte flow rates higher than 140 ml/min did not improve battery performance. In total, 40 experiments were executed representing different combinations of discharge current and electrolyte flow rate in the given range. Each dataset shows the discharge profiles of an individual run, as represented in Table 1. These datasets are available in the repositories^40^. The maximum standard deviation of the discharge profiles was 0.02 V. Table 3 shows the metadata description of each column in the dataset.

**Table 3:** Metadata of discharge and response test.

Data	Unit	Description
Current	mA	Measured current of the battery
Voltage	V	Measured voltage of the battery
Capacity	mAh	Calculated capacity of the battery
Specific Capacity	mAh/g	Calculated capacity of the battery incorporating the weight of zinc granules used
Energy	Wh	Calculated energy of the battery
Specific Energy	mWh/g	Calculated energy of the battery incorporating the weight of zinc granules used
Time	h:min:s.ms	Total operating time

## Technical Validation

For each experiment, a newly assembled battery with a fresh anode, cathode and electrolyte was used. However, a deviation in the data set became apparent, affecting battery capacity. This inconsistency characterizes a common phenomenon which must be considered for future large-scale production. Even though the deviation also influenced the voltage, its effect on the voltage was less than that on the battery capacity. In a previous study, the electrochemical compatibility of the materials used in the battery has already been confirmed^38^.

**Fig. 3:**
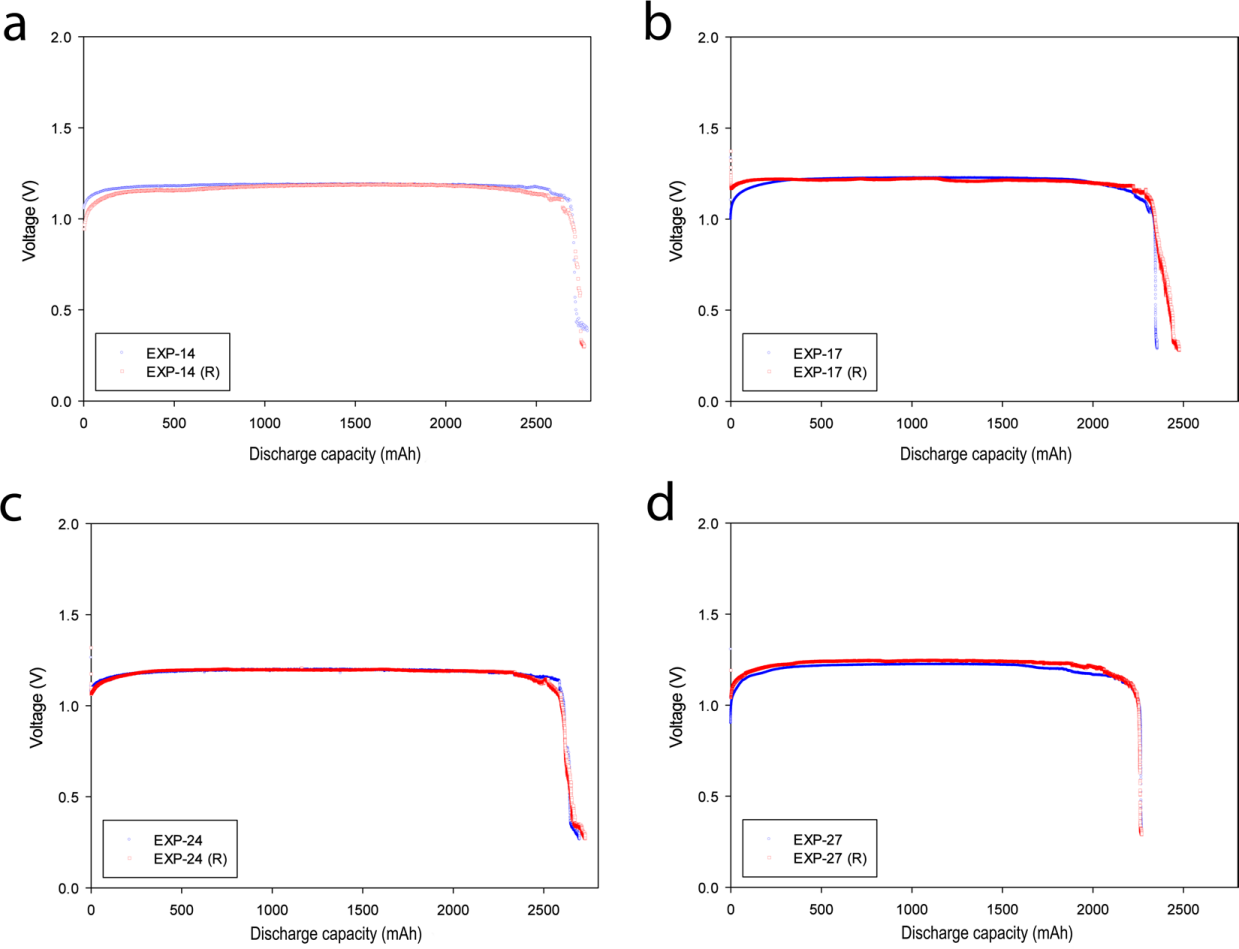
Discharge curves for the four different runs and the repeated experiment for each run: (**a**) electrolyte flow rate 60 ml/min and discharge current 175 mA (**b**) electrolyte flow rate 80 ml/min and discharge current 125 mA (**c**) electrolyte flow rate 100 ml/min and discharge current 175 mA and (**d**) electrolyte flow rate 120 ml/min and discharge current 125 mA.

To evaluate repeatability of the experiments, 20% of the total number of experiments (8 in total) were randomly selected and repeated. The dataset for each repeated experiment can be found under the name of EXP-X (R) (X shows the repeated experiment number). The repeated experiments, their specific capacity and energy, and their differences are represented in Table 4. Furthermore, Fig. 3 shows the discharge curves for the original and repeated experiments of four different runs: namely, EXP-14, EXP-17, EXP-24 and EXP-27. Thus, it is evident that the very low difference in values for both the original and repeated experiments as well as excellent agreement in the discharge curves of the original and repeated runs exhibited good repeatability, confirming the low error of the runs.

**Table 4:** Specific capacity and specific energy of original and repeated runs and their differences for 8 random experiments at cut-off voltage of 0.9 V.

Run	Specific Capacity (mAh/g)		Specific Energy (mWh/g)		Difference (%)	
	Original	Repeated	Original	Repeated	Specific Capacity	Specific Energy
EXP-5	564	589	660	663	4.2	0.5
EXP-13	490	486	590	569	0.8	3.6
EXP-14	541	542	637	630	0.2	1.1
EXP-17	469	471	564	568	0.4	0.7
EXP-24	524	522	619	618	0.4	0.2
EXP-25	560	560	660	664	0.0	0.6
EXP-27	453	451	542	551	0.4	1.6
EXP-29	559	568	672	662	1.6	1.5

## Usage Notes

The data provided here can be used in the model-based engineering of a ZAFB: either to fit the variables of an empirical model or to validate the results of a theoretical model. It is worth mentioning that the data was collected from an in-house developed flow battery, as described in Figs. 1 and 2. Battery behaviour could be unique for this kind of cell structure, depending strongly on the battery design and materials. Extra precautions need to be considered when comparing/extrapolating such data to data obtained from other batteries, having different designs, since such practice could be inaccurate.

## Data Availability

The data as reported were generated from experiments and are not relevant to any computer codes.
